# The mitochondrial genome of *Nemalecium lighti* (Hydrozoa, Leptothecata)

**DOI:** 10.1080/23802359.2021.1989335

**Published:** 2021-10-14

**Authors:** Jan-Niklas Macher, Ehsan Kayal, Elza Duijm, Berry van der Hoorn, Simone Montano, Arjen Speksnijder

**Affiliations:** a Naturalis Biodiversity Center, Marine Biodiversity, Leiden, The Netherlands; b Université de Caen Normandie, Biologie des Organismes et Ecosystèmes Aquatiques (BOREA), Caen, France; c Inholland University of Applied Sciences, Delft, The Netherlands; d University of Milano-Bicocca, Department of Earth and Environmental Sciences (DISAT), Milano, Italy; e University of Applied Sciences Leiden, Leiden, The Netherlands

**Keywords:** Mitogenome, hydrozoa, Leptothecata

## Abstract

The hydrozoan species *Nemalecium lighti* (Hargitt, 1924) is widely distributed in tropical marine waters around the world. Here we report the complete linear mitochondrial genome of *N. lighti* from Sint Eustatius (Lesser Antilles). The mitochondrial genome with a length of 14,320 bp encodes for 13 protein-coding genes, two tRNA genes, and two rRNA genes. Gene arrangement differs from that found in other species of the same taxonomic order and a phylogenetic analysis shows that based on mitochondrial genes, *N. lighti* clusters outside of the Leptothecata, rendering the order paraphyletic.

## Introduction

Hydrozoans are a diverse taxon with important functions in many aquatic ecosystems (Boero 1984; Puce et al. 2009) and are of interest for phylogenetic studies due to the evolution of linear mitochondrial genomes (Kayal et al. 2012). The species-rich hydrozoan order Leptothecata has recently been revised, and several new clades were identified based on phylogenetic analyses (Maronna et al. 2016). The species *Nemalecium lighti* (Hargitt, 1924), which is traditionally placed in the family Haleciidae, is of special interest due to its uncertain phylogenetic position based on ribosomal RNA (Maronna et al. 2016). We here report the complete mitochondrial genome of *N. lighti* from Sint Eustatius (Lesser Antilles, Caribbean Netherlands; 17.484 N, −62.997E). The analyzed specimen is stored in the Naturalis Biodiversity Center collection (accession number: RMNH.5017508; contact: collectie@naturalis.nl).

We extracted DNA with the Macherey-Nagel (Düren, Germany) NucleoSpin tissue kit on the KingFisher (Waltham, USA) robotic platform, and prepared a sequencing library using the NEBNext kit and oligos (New England Biolabs, Ipswitch, USA) following the manufacturer’s protocol. Sequencing was conducted on the NovaSeq 6000 platform at BGI (Shenzen, China). Read processing was conducted as in (Macher et al. 2017) with trimmomatic (v. 0.38 (Bolger et al. 2014)).

We assembled the reads using Megahit (Li et al. 2016) with the ‘meta-large’ function, annotated the mitochondrial genome using the MITOS server (Bernt et al. 2013) (translation table 4 (mold/protozoan/coelenterate; as reported in (Kayal et al. 2015)), and manually curated the annotations in Geneious Prime (v.2020.1) using previously published mtDNAs. The identification of the studied specimen as *N. lighti* was confirmed by mapping assembled contigs against 16S, 18S and 28S fragments of *N. lighti* downloaded from Genbank (Benson et al. 2017) (sequence accession numbers: KT757146.1; KT722410.1; KT266628.1 (Maronna et al. 2016) using the Geneious mapper with minimum 100 bp overlap and maximum 2% mismatch to the reference sequences allowed. We checked for potential contamination by other species by mapping reads against the *N. lighti cox1* and 16S with up to 45% difference allowed, but none was identified.

**Figure 1.**
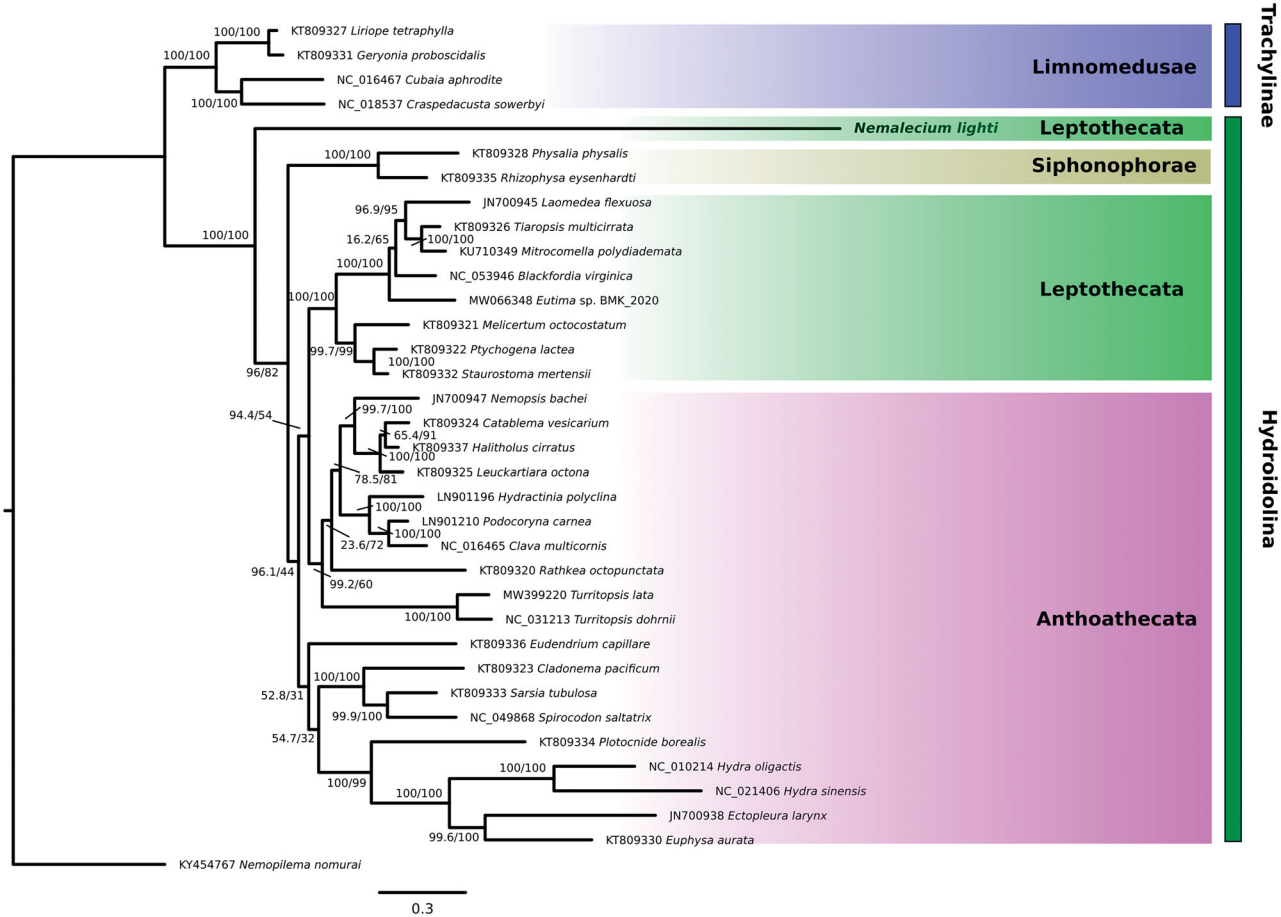
Phylogenetic tree showing evolutionary relationships of Hydrozoa based on 13 concatenated mitochondrial genes (3014 amino acids) from 34 Hydrozoa species. The scyphozoan Nemopilema nomurai was used as an outgroup. NCBI GenBank accession numbers are shown next to species names. The newly sequenced Nemalecium lighti is highlighted in bold. Numbers at nodes indicate bootstrap values (Maximum likelihood) and posterior probabilities (Bayesian inference). The tree was calculated using the IQ-Tree server with 1000 iterations of Ultrafast Bootstrap and visualised using FigTree (v1.4.4).

33 mitochondrial genomes of Hydrozoa species were downloaded from NCBI Genbank (accession numbers: see Figure 1; original studies (Kayal et al. 2012, 2015)) The scyphozoan *Nemopilema nomurai* Kishinouye 1922 (mitogenome downloaded from Genbank; study (Li et al. 2016)) was used as an outgroup. 13 protein-coding genes were translated into protein sequences and aligned using MAFFT. Gaps in the alignment were removed using Gblocks (Talavera and Castresana 2007). Sequences were concatenated and a phylogenetic tree was calculated using the IQ-Tree (Nguyen et al. 2015; Trifinopoulos et al. 2016) server (http://iqtree.cibiv.univie.ac.at/) with model selection using ModelFinder (Kalyaanamoorthy et al. 2017) and 10,000 ultrafast bootstrap replicates (Hoang et al. 2018) as commonly used for phylogenetic reconstruction based on mitogenomes (Kornienko et al. 2018; Deng et al. 2019; Li et al. 2019).

## Results

The assembled mitochondrial genome of *N. lighti* had a length of 14,320bp (base composition: A: 34.4%, C: 14.5%, G: 13.7%, T: 37.4%). 13 protein-coding genes, two tRNAs (trnW and trnM) and 2 rRNA (*rrnS* and *rrnL*) were recovered, as reported for other medusozoans. ATG and ATA are the main start codons except for *cox1* (CTG). TAA is the stop codon for all protein genes but *atp8*, *nad4L* and *nad5* (TAG). The mitogenome could not be circularized, meaning that it might be linear as reported for other hydrozoan taxa (Kayal et al. 2012). The *N. lighti* mitogenome showed a different gene order than other Leptothecata hydrozoans, but reminiscent of what has been described in aplanulatans (Kayal et al. 2015) (see figure deposited in figshare: https://doi.org/10.6084/m9.figshare.14866359.v1.)

The phylogenetic analysis of 13 concatenated protein coding genes from 34 hydrozoan species shows that *N. lighti* does not cluster within the Leptothecata, but as an outgroup of the other Hydroidolina, rendering the Leptothecata paraphyletic (see Figure 1). We point out that *N. lighti* was identified as a rogue taxon within the Leptothecata before and is treated as incertae sedis due to its unclear phylogenetic position based on 16S, 18S and 28S rRNA (Maronna et al. 2016). Our results show that the mitochondrial genome of *N. lighti* is potentially derived and that the phylogenetic position of the species might have to be reconsidered. However, the limited number of available mitochondrial genomes of Hydrozoa might lead to long-branch attraction and subsequent biased phylogenetic reconstruction. We refrain from drawing definitive conclusions and point out that more full mitochondrial genomes of hydrozoan species should be sequenced to understand the mitochondrial diversity and evolution within this taxon. Further, previous studies have shown that nuclear and mitochondrial phylogenies of cnidaria can disagree (Pratlong et al. 2017). Our findings on mitogenome rearrangement and phylogeny underline the uncertain phylogenetic position of *N. lighti* and should stimulate more research into this unique species. Further sequencing of additional non-aplanulatan mitogenomes might provide further deviations from the well-conserved genome organization in hydrozoans.

## Data Availability

The mitochondrial genome and annotation of *N. lighti* was deposited in figshare (https://doi.org/10.6084/m9.figshare.14899062.v1) and GenBank, accession number MZ457217. Raw reads are deposited in the Sequence Read Archive (SRA), accession number SRR10053108. The protein alignment used for calculating the phylogenetic tree is deposited in FigShare, doi: 10.6084/m9.figshare.14847348. A figure showing mitogenome topology of *Nemalecium lighti* and Leptothecata is available from figshare: https://doi.org/10.6084/m9.figshare.14866359.v1. The specimens RMNH.5017508 is availale from the Naturalis collection, contact: collectie@naturalis.nl.
